# The effect of nanoparticle coating on biological, chemical and biophysical parameters influencing radiosensitization in nanoparticle-aided radiation therapy

**DOI:** 10.1186/s13065-023-01099-7

**Published:** 2023-12-11

**Authors:** Elham Mansouri, Asghar Mesbahi, Hamed Hamishehkar, Soheila Montazersaheb, Vahid Hosseini, Saeed Rajabpour

**Affiliations:** 1 Student Research Committee, Tabriz University of Medical Sciences, Tabriz, Iran; 2 Drug Applied Research Center, Tabriz University of Medical Sciences, Tabriz, Iran; 3 Medical Physics Department, Medical School, Tabriz University of Medical Sciences, Tabriz, Iran; 4 Molecular Medicine Research Center, Institute of Biomedicine, Tabriz University of Medical Sciences, Tabriz, Iran; 5 Radiation Oncology Department, Olivia Newton-John Cancer, Wellness and Research center, Austin Health, Melbourne, Australia

**Keywords:** Coating layer, Coating materials, Metal nanoparticles, Nanoparticle surface, Radiosensitization, Radiation therapy

## Abstract

Nanoparticle-based composites have the potential to meet requirements for radiosensitization in both therapeutic and diagnostic applications. The radiosensitizing properties of nanoparticles could be reliant on the nature of their coating layer. Any gains in reduced toxicity and aggregation or improved delivery to tumor cells for coated nanoparticles must be weighed against the loss of dose enhancement. The radiosensitization potential of coated NPs is confirmed by numerous studies but in most of them, the coating layer is mostly applied to reduce toxicity of the NPs and for stability and biocompatibility aims. While the direct effects of the coating layer in radiosensitization—were ignored and not considered. This review provides an overview of double-edged impact of nanoparticle coating on the radiosensitization potential of nanostructures and discusses the challenges in choosing appropriate coating material in the aim of achieving improved radioenhancement. Coating layer could affect the radiosensitization processes and thereby the biological outcomes of nanoparticle-based radiation therapy. The physicochemical properties of the coating layer can be altered by the type of the coating material and its thickness. Under low-energy photon irradiation, the coating layer could act as a shield for nanoparticles capable of absorb produced low-energy electrons which are important levers for local and nanoscopic dose enhancement. Also, it seems that the coating layer could mostly affect the chemical process of ROS production rather than the physicochemical process. Based on the reviewed literature, for the irradiated coated nanoparticles, the cell survival and viability of cancer cells are decreased more than normal cells. Also, cell cycle arrest, inhibition of cell proliferation, DNA damage, cell death and apoptosis were shown to be affected by coated metallic nanoparticles under irradiation.

## Background

**Fig. 1:**
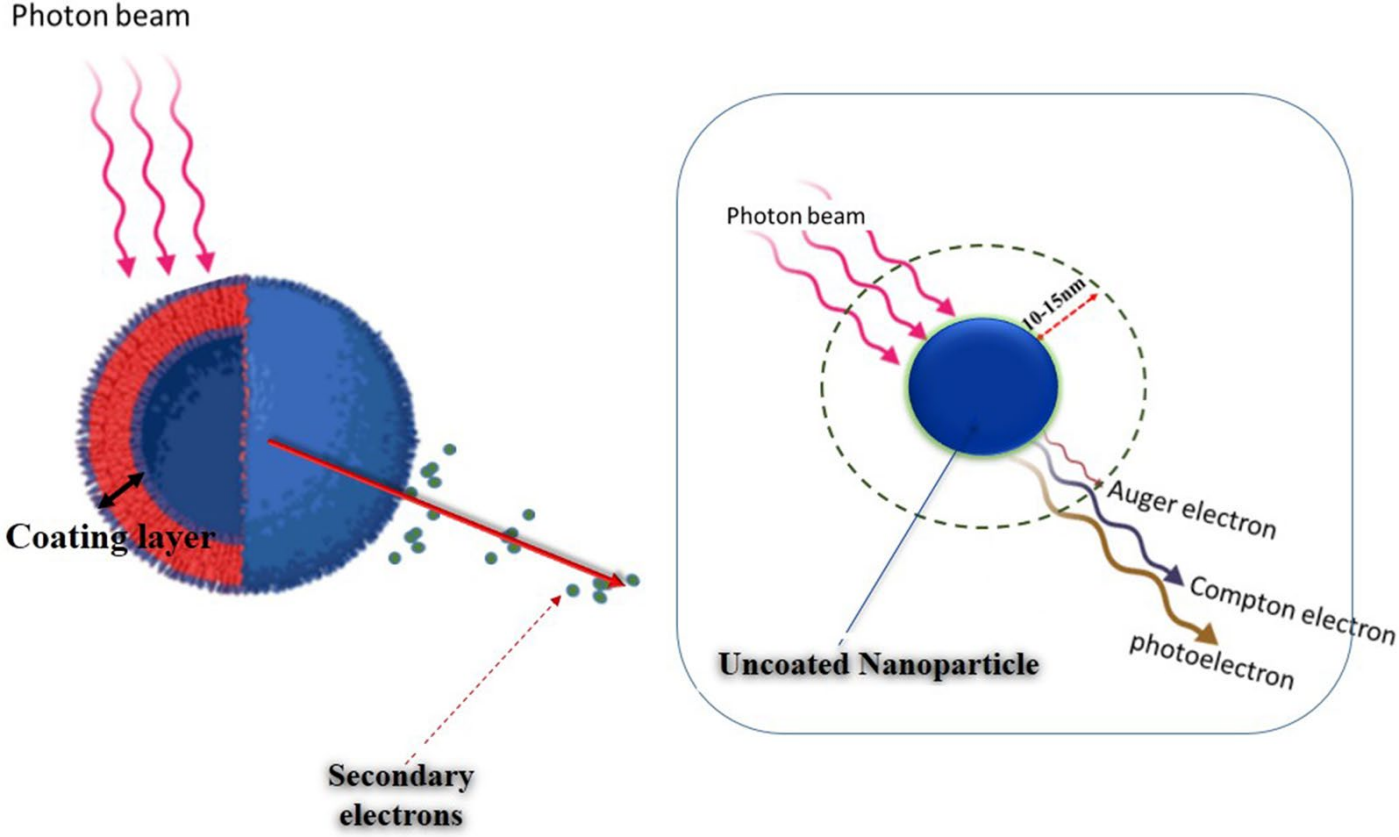
Effect of coating layer on radaiosensitization processes

Many efforts in radiation therapy have focused on approaches that aim to preferentially radiosensitize tumors whilst minimizing side effects in adjacent normal tissues. In radiotherapy, one of the major challenges is the lack of selectivity due to the similar mass energy absorption properties of cancerous and healthy tissues [1]. The application of radiosensitizers in radiation therapy is an effective way to boost the tumor-killing efficacy of radiotherapy with lower doses of radiation. Radiosensitizers can enhance the effects of radiation therapy via multiple mechanisms including physical, physico-chemical, chemical and biological processes [2–9]. Due to recent advances in nanotechnology, it is increasingly possible to selectively accumulate metal nanomaterials in tumor cells to enhance the contrast between tumor and normal tissues, leading to enhanced radiosensitizing properties compared to radiotherapy alone [6]. The efficacy of radiosensitization with metallic NPs is based on the generation of secondary electrons, free radicals, and reactive oxygen species (ROS). Generally, NPs consist of a core generated from various synthesis techniques to give rise to a wide range of sizes and shapes that is then covered by a surface coating. Surface coating of NPs can be functionalized for a variety of applications such as imaging, drug delivery, diagnosis and therapy. The radiosensitizing properties of nanoparticles could possibly be reliant on the nature of their coating layer and physico-chemical properties of them can be altered by the type of the coating material and its thickness. The radiosensitization potential of coated NPs is confirmed by numerous studies but in most of them the coating layer is mostly applied for reducing toxicity of the NPs and for stability and biocompatibility aims. While, the direct effects of the coating layer in radiosensitization was ignored and not considered. Nanomaterials could be modified with various coatings which could modulate their behavior in biocompatibility, stability and toxicity. The coating layer could affect the radiosensitization processes and thereby the biological outcomes of NP-based radiation therapy (Fig. 1). This review provides an overview of double-edged impact of nanoparticle coating on radiosensitization potential of nanostructures and discusses the challenges in choosing appropriate coating material in with the aim of achieving improved radioenhancement. Several studies are conducted to evaluate the effect different coating materials on radiosensitization properties of nanoparticles (Table 1). In this review, we attempt to bring together the current data on the effects of NP coatings on the radiosensitization potential of nanostructures and describe the effects of coating layer on radiosensitization process including physical, physicochemical, chemical and biological. Also, the effect of coating layer on biological consequences of nanoparticle-based radiation therapy such as cell survival and viability, inhibition of cell proliferation and DNA repair and cell death were discussed. This review could provide a theoretical basis for the future development of nanoparticle-based radiotherapy and design of nano-radiosensitizers.

**Table 1:** Coated nanoparticles in radiosensitization studies

Study	Irradiation properties	Coating layer-nanoparticle (NP)	Finding
Li et al. [57]	–	PEG-coated ceria NP	Reduced liver cell uptake over uncoated ceria
Kong et al. [14]	200 kVp X-rays	Glucose-coated gold NP	Increased MCF-7 cell uptake comparing to non-coated NPs
Song et al. [56]	–	Glucose-coated gold NP	Increased HeLa and MCF-7 carcinoma cells uptake comparing to non-coated NPs
Zhang et al. [59]	–	PEG-coated gold NP	Increased HepG2 cells uptake comparing to non-coated NPs
Kong et al. [14]	200 kVp X-rays	Glucose-coated gold NP	Increased cell death comparing to the non-coated NPs
Klien et al. [131]	Low dose X-ray	Citric-coated iron oxide NP	Increased ROS generation comparing to the non-coated NPs
Peukert al. [95]	5 and 10 MeV proton	PEG-coated gold NPs	Large loss of enhancement occurred for thick nanoparticle coatings
Fathy et al. [26]	0.5, 1, 2, 4 Gy electron	Silica-coated iron oxide NP	Silica coating layer do not destruct the superparamagnetic behavior of iron oxide NPs
Nakayama et al. [18]	150 kVp	PAA-Titanium oxide NP	Increased ROS generation comparing to the non-coated NPs
Fathy et al. [26]	0.5, 1, 2, 4 Gy electron	Silica-coated iron oxide NP	Increased ROS generation comparing to the non-coated NPs
Gilles et al. [112]	X-ray	PEG-coated gold NP	Reduced ROS generation comparing to the non-coated NPs
Singh et al. [97]	X-ray	Silica-coated iron oxide NP	Reduced ROS generation comparing to the non-coated NPs
Zhang et al. [21]	6 MeV)	PEG-coated gold NPs	Clonogenic survival curve was linearly decreased in the presence of coated gold NPs and non-linear in the absence of NPs
Chithrani et al., [125]	6 MeV X-ray	Galactose-PEG-coated gold NP	Survival fraction decreased by an increase of radiation dose (0–8 Gy 6 MeV) in the presence of galactose-PEG-coated gold NPs was more than that of uncoated gold NPs
Spass et al. [105]	X-ray	PEG-coated gold NP	PEG shell can substantially alter the spectra of low-energy secondary electrons escaped from the PEG-coated gold NP surface
Belousov et al. [75]	8 keV to 1 MeV X-ray	PEG-coated gold NP	Surface grafting of gold NP with PEG can change the amount and the energy spectra of secondary electrons generated within the irradiated gold core

## Coated nanoparticles as radiosensitizers in cancer therapy

Nanoparticles were shown to have the ability to enhancing tumor targeting and tumoricidal effects of the radiation. The radiosensitizing properties of nanoparticles could be reliant on the nature of their coating layer. Generally, physicochemical properties of metallic nanoparticles can be altered by the type of coating material and the layer thickness. Nanoparticles have shown great promise as radiosensitizers in preclinical studies. Radiosensitizing effect of glucose-coated gold NPs was seen on lung cancer cells [10], ovarian cancer cells [11] and human prostate cancer cells [12]. A study by Hainfeld et al. [13] showed a significant dose enhancement effect for glucose-coated gold NPs combined with 250-kV X-rays in mice with mammary carcinomas. As Roa et al. [12] reported, glucose-coated gold nanoparticles followed by 2 Gy of ortho-voltage irradiation reduced prostate cancer cell growth by 1.5–2.0 fold compared with naked gold NPs. They also demonstrated that glucose-coated gold NPs enhanced only the radiation sensitivity of cancer cells (DU-145), while having no observable effect on non-malignant fibroblasts (MRC5). Moreover, Kong et al. [14] showed that glucose-coated gold NPs enhance the radiation sensitivity in breast-cancer cells but not in nonmalignant cells. Silver NPs with multiple different coatings have also been shown to exhibit higher anticancer efficacy against Glioma cell lines when combined with ionizing radiations [15]. In a study by Fagundes et al. [16], they showed that citrate-coated cobalt and nickel ferrite NPs increased therapeutic efficacy of radiotherapy by 2.5 to threefold. The study by Zhang et al. [17] demonstrated that tumor inhibition by radiotherapy can be significantly improved by using glutathione-coated gold nanoclusters as radiosensitizers. The most common strategies to enhance distribution, prevent aggregation, and reduce the toxicity of nanoparticles include coating the particles with polymers such as polyethylene glycol (PEG), and changing the size and shape of the nanoparticles. It was reported that polyacrylic Acid (PPA)-coated titanium oxide NPs inhibited growth of tumors when applied in combination with X-ray radiation [18]. Several line of studies showed increased therapeutic efficiency of polyethylene glycol (PEG)-coated gold NPs for radiosensitization [19–21]. Studies indicated that PEG-coated gold NPs had greater therapeutic efficacy than nude NPs [19–21]. Dou et al. [22] reported significant effects of PEG-coated gold NPs on inhibiting tumor growth under 6MV irradiation. Zhang et al. [21] indicated that PEG-coated gold NPs could decrease the tumor volume and weight after 5 Gy radiation. In other study, a significant delay in tumor growth under 6MV electron beams was reported by Cheng et al. [23] for Citrate-coated gold NPs. Glucose-coated gold NPs can enhance efficacy of X-ray treatment by 30.4 ± 13:5% at low X-ray dosage under the clinically used irradiation energy of 6MV photon beam [24]. Radiation resistance is one of the major causes of radio therapy failure and subsequent tumor relapse which is related to the hypoxic tumor microenvironment. Yi et al. [25] showed that gold NPs coated with MnO_2_ could help to overcome hypoxia-associated radiation resistance leading to more radiosensitization efficiency of gold NPs. Fathy et al. [26] indicated the silica-coated iron NP as a promising radiosensitizer in breast cancer radiotherapy. Durand et al. [27] Showed that gadolinium chelate-coated gold NPs could decrease aggressiveness and invasiveness in glioblastoma.

## Biocompatibility and stability

The surface coating is an important parameter in controlling particle toxicity, stability, solubility and biocompatibility. Biocompatibility describes the ability of a material to appropriately perform its desired function without causing any toxicity or adverse immunological response in living tissue or a living system [28]. Uncoated NPs are significantly toxic both in-vitro and in-vivo, and appropriate coating may lessen this adverse effect. In the case of metallic NPs, especially gold NPs, citrate coating is the most commonly used coating material. However, the citrate-coated metallic NPs have a high zeta potential which makes them easy to aggregate [29]. To overcome this problem, a variety of alternative surface coatings are used to make the NPs more biocompatible, either replacing or augmenting the citrate capping. Several in-vitro and in-vivo studies confirmed important effect of PEGylation on NP biodistribution, stabilization, and structure [20, 30–32]. It was reported that the safe dose of the PEG-coated gold NPs is about 10^4^M [33, 34] and cytotoxic effects of the PEG-coated gold NPs are increased in a clear concentration-dependent manner [21]. Kumar et al. [35] demonstrated the biocompatibility and low cytotoxicity of PEGylated gold NPs in Hela cells. Moreover, uncoated silver NPs were found to be more toxic than coated ones [36]. One of the important factors determining the accumulation of NP within the tumor is its circulation time in bloodstream. Keeping NPs in bloodstream for a sufficiently long period is a major challenge because NPs are quickly opsonized and engulfed by the macrophages, thereby shortening their circulation times. Surface modification improves the circulation time by preventing NP engulfment by the mononuclear phagocyte system (MPS) and subsequent removal from circulation [37]. One possible approach to prolong the circulation time of NPs in the bloodstream is to modify them with PEG [38]. PEG molecules form a well hydrated inert hydrophilic layer on the surface of the NP preventing other molecules to bind the NP surface via steric repulsion forces [39]. It is demonstrated that PEG-coated gold NPs are less likely to be uptaken by macrophages [40]. Moreover, it is reported that the uptake of the magnetic NPs by macrophage cells is greatly reduced after coating with PEG. Importantly, the surface charge of metallic NPs plays a cardinal role in their stability (eg, tendency to aggregate) in aqueous solution and in the body [41]. Applying coating provides several benefits for introducing controlled charges on the surface of the NPs [42]. Silica coating is one of the most popular techniques for nanoparticle surface modification [43]. It can be used as a coating material for metallic NPs for radiosensitization such as gold [44, 45], iron oxide [46] or multicomponent cores [47]. TEM images prove that the silica coating layer can act as a supporting substrate that prevents iron oxide NPs from aggregation [26]. Fathy et al. [26] reported that the presence of silica coat could increase the biocompatibility of the iron oxide NPs without any negative effect on the radiosensitization. Also, they demonstrated that the silica coating layers enhance the stability of iron oxide NPs by increasing the surface negative charge. [38].

## Cellular uptake and internalization

Although non-functionalized NPs have the potential to attach to and be partially uptaken by cancer cells, it is desirable to synthesize NPs with suitable functional groups for efficient targeting and internalization [48]. Generally, surface coating of NPs could influence the radiosensitization properties by increasing cellular uptake and accumulation in tumor cells more than in normal cells leading to generation of a significant contrast between tumor and normal tissues in terms of radiosensitivity. Surface modifications help to optimize the biodistribution of NPs and can be used to enhance the cellular uptake and determine NP sub-cellular localization [49]. Zeta potential can influence the cellular uptake and intracellular trafficking of nanomaterials. The uptake of positively charged NPs in different cell lines was superior to that of negatively charged counterparts [50]. More uptake and accumulation of NPs by tumor cells will cause stronger photoelectric interaction and more secondary electrons to be produced, which will result in greater tumor control. PEG-modified gold NPs offer distinct advantages, including less cytotoxicity and enhanced cell uptake, and give valuable insights into further surface modifications of therapeutic NPs [21]. Chithrani et al. [30] showed that spherical NPs are uptaken more efficiently than other shapes such as cubic and rod NPs. It has been demonstrated that the roughness of NPs surfaces enhances the binding and cellular uptake due to increasing of its surface area. In another study, Fathy et al. [26] implied that the surface roughness of silica-coated iron oxide NPs is about 1.3-folds greater than that of iron oxide NPs, leading to more cellular internalization of silica-coated iron oxide NPs. Glucose-coated gold NPs which are designed based on cancer cell metabolism, can be selectively taken up by cancerous cells and accumulate in the cytoplasm [10, 14]. Zavestovskaya et al. [51]indicated that coating of nanoparticles with polymers such as PEG could help NPs accumulation in solid malignant tumors due to an inherent leakiness of the neovasculature of the growing tumor. Increased cellular uptake of citric- or malic-coated iron oxide NPs was reported by Klein et al. [52]. Cell internalization is increased by glucose, resulting in an increase in radio sensitivity [53]. The targeting ability of glucose-coated gold NPs has been demonstrated in various solid tumors such as breast, lung, and ovarian cancers [12, 14, 54, 55]. Glucose-coated gold NPs designed based on cancer cell metabolism, can be selectively taken up by cancerous cells and accumulate in their cytoplasm [12, 14]. According to Kaur et al. [48], glucose-coated gold NPs show a sevenfold increase in cellular uptake in DU-145 cells compared to gold NPs without glucose binding, suggesting that glucose plays a crucial role in the delivery of more gold NPs into cells. Song et al. [56] reported a reasonably rapid enhancement of glucose-coated gold NPs uptake by HeLa and MCF-7 carcinoma cell lines comparing the uptake of naked gold NPs. They also indicated that the cellular uptake of metallic NPs depends on cell type. Kong et al. [14] showed that glucose-coated gold NPs had a significant increase in cellular uptake in MCF-7 cells with glucose-coated gold NPs compared to the uncoated ones. Besides, Li et al. [57] reported that PEG-coated ceria NPs reduced liver cell uptake over uncoated ceria NPs. As a result, unhealthy cells are more effectively killed than healthy ones [20]. An in-vitro study showed that PEGylated (functionalized with PEG) gold NPs enhanced cellular uptake in B16F10 murine melanoma cells, resulting in radiosensitization under irradiation with 6 MeV [58]. According to Zhang et al. [59], galactose-PEG-coated gold NPs increased cellular uptake three-fold compared with uncoated gold NPs in HepG2 cells. It seems that coating could enhance localization in the site of interest and the selective sensing properties of NPs. In addition to being biocompatible, Poly lactic-co-glycolic acid (PLGA)-coated iron oxide NPs have the advantage of penetrating into cells [60]. It is well documented that folate is suitable as a targeting agent due to its stability, non-immunogenicity, and specificity for cancer cells, as well as its simple conjugation chemistry [61, 62]. Kefayat et al. [63] showed that folate- and BSA-coated gold NPs accumulate in C6 Glioma tumor cells 2.5 times higher than that in normal cells, demonstrating their excellent targeting ability. Khoshgard et al. [61] found that folate-conjugated gold NPs had higher internalization ability in Hela cells, as well as higher cancer cell death rates and dose enhancement factor (DEF) under different irradiation energies when compared to PEGylated gold NPs. The in-vitro study by Liu et al. [64] revealed that iron oxide NPs coated with amino group (NH2-NanoMag) could take up by DU145 cancer cells, localized in cytoplasm, and cause radiosensitization upon exposure to MV X-ray beams.

## Physical enhancement

### Effect of photon beams

**Fig. 2:**
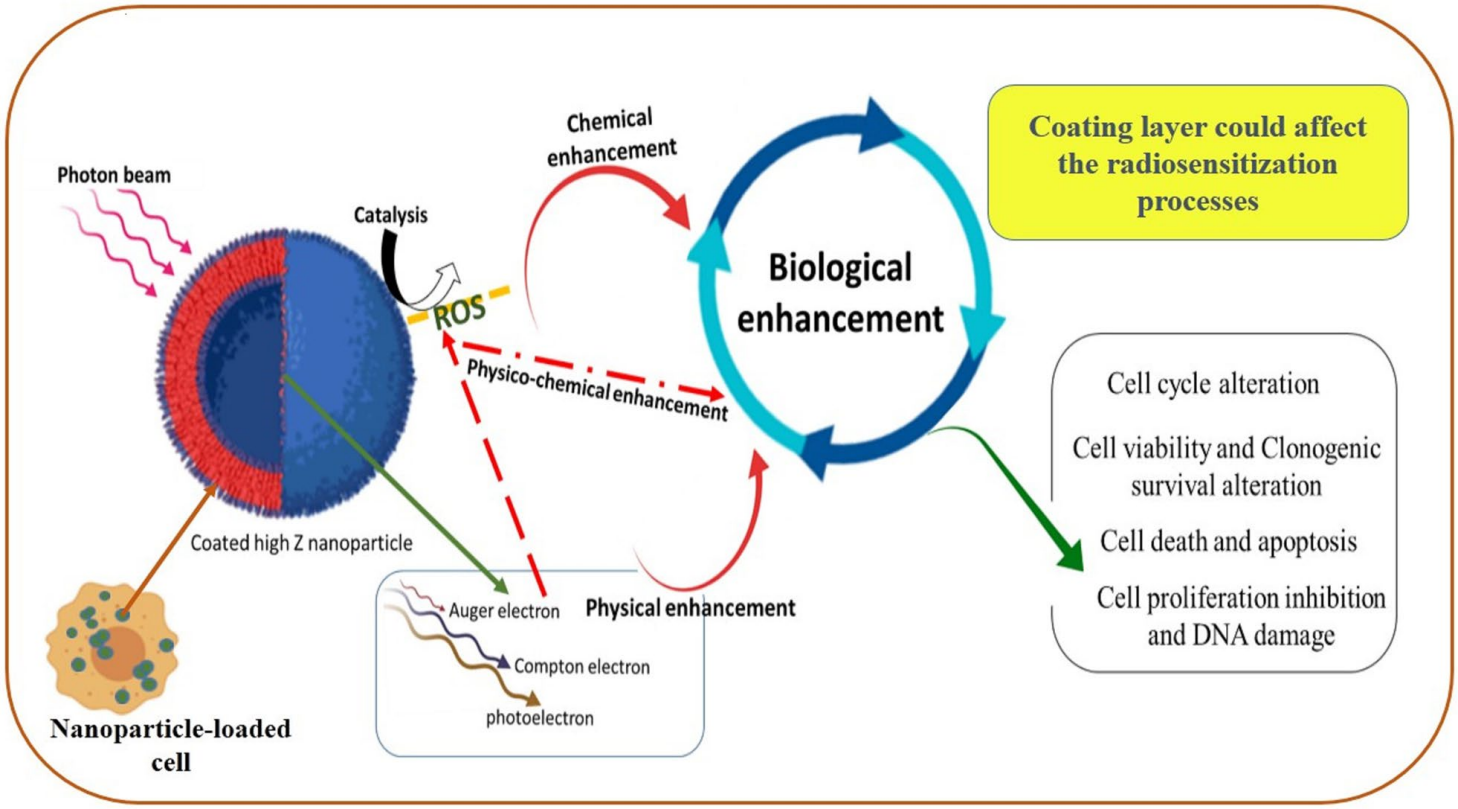
Effect of coating layer on secondary electron production

A photon's interaction with NPs causes transference of electromagnetic energy from the incident photon to the material via Compton, photoelectric or pair production interactions, resulting in a local dose deposition around NPs. Subsequently, Secondary diffusion of energy from NPs happens via the generation of photoelectrons and Compton electrons together with pair production and Auger electron cascades depending on the energy of initial photon [65]. Nanodosimetric characteristics of NPs suggest that both kilo-electron-volt (keV) photons and clinical megavoltage (MV) sources increase biological damages [66, 67]. Based solely on physical dose enhancement, the radiosensitization of NPs is significant at keV energy levels, but not at MV levels [68, 69]. It seems that biological consequences of NP for keV photons is mostly related to the physical effect of NPs. While for MV photons, the chemical effects are mainly involved in radiosensitization process. It is well known that for low-energy keV photons, radiosensitization has mainly been attributed to physical dose enhancement via photoelectric interaction. Low-energy photons have a large cross-section of photoelectric interaction on the K-, L- and M-shells of the materials. Meanwhile, for photon beams near the K-shell of targeted NPs, low-energy short-ranged Auger cascade is more likely to be generated which could enhance nanoscopic energy deposition. Because of short-range of these electrons, the NPs must be proximal to the biological target (DNA) to impart an effect by physical mechanism. In this case, low concentration of high-Z NPs could result in significantly more energy deposition per unit of mass in tumor than the surrounded soft tissue. Actually, under low-energy photons, the coating layer could act as a shield for NPs capable to absorb produced low-energy electrons which are important levers for local and nanoscopic dose enhancement. Auger electrons account for the largest share of produced secondary electrons. These low energy electrons has short range about 10–15 nm and could be stopped by the coating layer. While, photoelectrons and Compton electrons have longer range and could escape from the coated nanoparticle surface (Fig. 2). Several Monte Carlo studies have shown that most of the dose is deposited within the first few nanometers surrounding the nanoparticle due to the high-Z nanoparticles enhancement under low-energy photons [69, 70]. Due to the low atomic number of common coating layers and the dominance of photoelectric effect in this energy range, there is little expectation for the coating layer to interact with the photon beams and involve in physical dose enhancement. Low energy short-range Auger electrons have high LET (linear energy transfer) and deposit their energy in a short distance from place they generated. Therefore, Auger electrons are important for increasing local dose distribution. In addition spectroscopy of metallic nanoparticles showed that large number of secondary electrons generated from photon and nanoparticle interactions, are Auger electrons [6, 71]. These electrons could induce single strand breaks and double strand breaks to the DNA molecules directly or could indirectly increase ROS production leading to DNA damage and cell death [72]. So the coating layer could affect the number of Auger electrons reach to the tumor cells in the vicinity of metallic nanoparticles and influence the radiosensitization properties of nanoparticles Due to the low atomic number of common coating layers and the dominance of photoelectric effect in this energy range, there is little expectation for the coating layer to interact with the photon beams and involve in physical dose enhancement. The dose enhancement of MV photons is lower than kV photons, but still far greater than the values reported by MC simulation, which is due to the chemo-biological effect of NPs. Due to the predominance of the Compton or pair production effect for high-energy photons, a very sparse distribution of ionization events occur when high-energy photons are present, and in this case negligible physical dose enhancement effect is expected. The use of photon sources above the k-edge of targeted NPs leads to production of long-ranged particles depositing their energy far from the NP and low-energy electrons are less expected to be generated. In this condition, a higher concentration of NPs in the tumor region is required to achieve acceptable physical dose enhancement. In the case of clinically relevant MV energies, NPs display a much greater radiobiological response than can be justified only based on physical dose enhancement [69, 73]. Accordingly, for this range of energy levels, chemical properties of NP surfaces could activate mechanisms such as increased ROS generation, cell cycle arrest, and DNA repair inhibition and so on, contributing to chemical and biological dose enhancement. Thus, it seems that surface coating and functionalization of NPs play an important role in chemo-biological dose enhancement in high-energy range irradiation.

Under low-energy photons, the coating layer could act as a shield for NPs capable to absorb produced low-energy electrons which are important levers for local and nanoscopic dose enhancement. For this reason, the thickness of the coating layer must be taken into account in the design of nano-radiosensitizers. Simulation studies showed that under low-energy photon irradiation, PEG shell can substantially alter the spectra of low-energy secondary electrons escaped from the gold NP surface [74–77]. Xiao et al. [78] suggested that coatings may considerably diminish the short-range low-energy electrons emitted from gold NPs, thereby radiosensitization decreases considerably. Irradiation of brain tumor in the presence of PEGylated gold NPs by 175 kVp X-ray photons resulted in a twofold elongation of animal lifespan [79]. Soleymanifard et al. [55] showed that glucose-coated gold NPs have remarkable potential for radiosensitization of MCF7 and QU-DB cells under exposure of both low and high- energy photon beams. Her et al. [80] reported DEF of 1.26 and 1.15 for MDA-MB-231 and MDA-MB-436 cell lines loaded with PEG-coated gold NPs and irradiated by MV clinical photons. Khoshgard et al. [61] showed that for the Hella cells loaded with folate-coated gold NPs, the DEFs of all the orthovoltage (120 to 250 kVp) beams were greater than those of the Co-60 gamma-rays. Rezaei et al. [81] reported that using the dextran-coated iron oxide NPs in the presence of MV electron beams can increase the radiosensitivity of HeLa and MCF-7 cancerous cell lines. Wang et al. [10] demonstrated a significant radiosensitization effect of glucose-coated gold NPs in A549 cells irradiated by 6MV X-rays with a sensitivity enhancement ratio (SER) of 1.5, suggesting that combining glucose-coated gold NPs and radiation therapy may have therapeutic potential in lung adenocarcinoma. It was demonstrated that iron oxide NPs with the appropriate surface modifications have been shown to enter DU145 cells and can act as a cell sensitizer to megavoltage ionizing radiations in radiation therapy [64]. Kong et al. [14] demonstrated that under 200 kVp X-rays, functionalized thioglucose-coated gold NPs increased cell death significantly in breast cancer cells compared to naked gold NPs. Kaur et al. [48] indicated that the presence of glucose-coated gold NPs in the Hela cells irradiated with 60Co source (1173 keV and 1332 keV photons), produced more Compton electron leading to higher radiolysis and DNA damage. They also demonstrated a 29% dose reduction for NP-treated Hela cells compared to cells without glucose-coated gold NPs. Using 500–1000 keV photons, Belousov et al. [75] reported that coated gold NPs produced up to 20% more Compton electrons than uncoated NPs, leading to a higher total number of secondary electrons within gold NPs.

It seems that under high-energy photon beams, the surface coating of NPs is mostly effective in chemo-biological enhancement and less in physical enhancement. For high-energy photons by domination of Compton Effect or pair-production, long-ranged particles are expected to be generated from the NP. Commonly, these particles are not stopped by the coating layer and deposit their energy far from the NP. Meanwhile, short-ranged low-energy secondary electrons are mainly absent in this energy range and hence nanoscopic dose enhancement is not expected. On the other hand, the coating layer may interact with the high energy photon beams resulting in production of Compton electrons, scattered photons and other secondary particles but the resultant physical dose enhancement is insufficient to adequately explain observed biological responses. It could be suggested that under high-energy photons irradiation, higher concentration of coated NPs should be applied to amplify physical dose enhancement. ROS production capacity of metallic NPs and act as a radioprotective agent for normal cells. Abidini et al. [82] reported PEG-coated bismuth nanoparticles as an effective radiosensitizer in radiation therapy under megavoltage beams. Jafari et al. [83] suggested that combination of PEG-coated iron nanoparticles with 6MV X-ray could reduce survival of U87-MG cells. Radiosensitization of breast cancer to mega-voltage radiation therapy with PEG-coated gold-iron nanoparticles was increased under MV beams of 18 MV [84]. Mohammadian et al. [85] suggested PEG-coated iron nanoparticles as promising nano-radiosensitizer in colorectal cancer treatment under 6-MV X-ray photons. Geng et al. [11] demonstrated that glucose-coated gold NPs achieved superior enhancement ratios at 90 kVp than 6 MV. Based on their results, the DEF value for glucose-capped gold NPs was reported up to 1.44 for 90 kVp and up to 1.37 for 6MV. Wang et al. [54] reported the radiosensitization effect of thioglucose-coated gold NPs on triple-negative breast cancer cells irradiated by 6MV photons. DEF values of 1.33–1.59 have been reported by Liu et al. [19] for PEGylated gold NPs loaded in CT26 cells irradiated by 6 MV photons. The results of study by Kirakl et al. [86] raise the possibility that effects of citrate-coated iron oxide NPs may cause dose-dependent and cell line specific radiosensitization at 6MV X-ray energies. Wang et al. [87] reported Glucose-coated gold NPs as a new radiosensitizer, combined with radiation, can increase cytotoxicity on A549 cells not only by arresting the G2/M phase and by increasing apoptosis probably.

### Particle therapy

Comparing to the X-rays, ion beams have densely ionizing feature causing larger relative biological effectiveness (RBE) which provide more cancer killing efficacy[88]. The radioenhancement effect of metallic nanoparticles as potential radiosensitizers in particle therapy has been recently reflected in both simulation and experimental radiobiology studies [89]. Charged particles usually lose energy in a large number of subsequent ionization and excitation interactions with atoms of the medium they pass through. Due to the larger surface/valium ratio of nanoparticles comparing to the bulk material, presence of nanoparticle could increase the interaction of the particles with matter leading to more secondary particles and ROS generation providing more radiation induces damages. Metallic NPs could influence the damaging effect of particle therapy mostly by chemical effect and ROS generation. The coating layer could affect the amount of ROS generation [26]. Porcel et al. [90] demonstrated the amplification effect of polyacrylic acid-coated platinum NP under medical carbon beams highlighting the role of ROS in radiation damage. Zwiehoff et al. [91] indicate that proton irradiation-induced ROS formation sensitized by noble metal NPs is driven by the total available particle surface area rather than particle size or mass. The result of study conducted by Fathy et al. [26] showed that coating of iron NPs with silica could enhance the therapeutic efficacy of electron therapy in breast cancer treatment. Rezaei et al. [81] indicated that dextran-coated iron NPs can increase radiosensitivity and consequently at a given absorbed dose could lead to more cell killing will occur in cancerous cells leading to improve of the efficiency of electron therapy. Li et al. [92] found that 2 MeV protons have greater tumor killing effect on epidermoid carcinoma cells (A 431), when combining with PEG-coated gold NPs. Cunningham et al. [93] Suggested that citrate-coated gold NPs could increase dose enhancement in proton therapy. Compared to the naked bismuth oxide NPs, the PEG-coated bismuth oxide NPs are found more effective in increasing the treatment efficacy of electron therapy Zavestovskaya et al. [51] reported enhanced proton therapy for PEG-coated boron NP for cancer therapy. Jeynes et al. [94] reported that citrate capped gold nanoparticles do not amplify the effects of 3 MeV protons on RT112 bladder cancer cells. A simulation study by Peukert al. [95] suggested that under proton beams, coating layer should be kept the minimum to reduce the loosing of dose enhancement. Also, they indicated that denser coating layer may have a minimal effect on increasing dose enhancement.

## Effect of coating on magnetic properties of magnetic nanoparticles

Magnetic NPs are considered as one of the most promising substances for cancer therapeutic and diagnostic applications. Magnetic nanocomposites with a core–shell structure have huge applications in medicine [96]. However, in order to promote dispensability in aqueous solutions and biocompatibility, magnetic NPs must have surface modifications [97]. Silica may offer significant advantages as a coating shell for magnetic NPs in terms of water-solubility and biocompatibility [98–100]. Relying on this concept, Alwi et al. [101] reported the benefits of using silica-coated superparamagnetic iron oxide NPs as contrast agents in biomedical photoacoustic imaging. Moreover, Fathy et al. [26] indicated that silica coating layer do not destruct the superparamagnetic behavior of iron oxide NPs and the composite can be easily recovered by magnetic separation. The thick-coating layer has often been shown to have deleterious effects on NPs magnetic properties and thus their potential applications [97, 102, 103]. Singh et al. [97] showed that decreasing the silica-layer thickness of the silica-coated iron oxide NP makes the zeta potential decline toward negative values resulting in an increase in magnetic properties of the NP.

## Chemical and physico-chemical responses of coated NPs

### ROS generation

The physical mechanisms involved in radiosensitization are insufficient to adequately explain observed biological responses. Thus, a better understanding of other mechanisms underlying these effects is required. Physico-chemical and chemical mechanisms are important to exert indirect damage to cancer cells via a pronounced increase in free radicals and ROS generation. The formation of free radicals and nonradicals of reactive oxygen species (ROS) could trigger antimicrobial and anticancer functions for metallic nanoparticles leading to damage of cell membrane [104]. ROS are the byproduct of secondary electrons interaction with the aquatic environment (physico-chemical process) and/or produced via chemical interaction of NP surface with biological system (chemical process). Although the role of ROS in the cancer treatment is controversial, the evaluation of their production could bring new information concerning the radiosensitizing effect of the studied NPs. ROS modulation is an indispensable factor in getting suitable therapeutic result due to the fact that the excessive production of ROS is the most common side effect of metallic nanoparticles, which can lead to NP-induced toxicity [104]. The combination of ROS-generating agents with radiation exposure is likely to cause cytotoxicity in an additive or synergistic manner. Controlling the amount of ROS and specific targeting is for achieving promising anticancer and antimicrobial results in physiological condition [104]. In the presence of hydrogen peroxide or molecular oxygen, metallic NPs undergo Haber–Weiss and Fenton redox reactions to form hydroxyl superoxide radicals (chemical mechanism) [8, 105]. The localization of radiation-induced damages and increased levels of ROS within cells can overwhelm the antioxidants and disrupt redox equilibrium, triggering oxidative stress, biomolecular damages and cell death [106, 107]. Meanwhile, electrons (as byproducts of radiation interaction with matter) interact and ionize oxygen-containing molecules in the vicinity of the NP, thereby generating ROS [108]. Production of ROS is likely to be dependent on many variables and characteristics of the metal NP, such as size, shape and surface chemistry. The surface properties of NPs such as their interfacial layers, coatings, functionalization, and/or capping agents can play a significant role in ROS generation [109–111]. Haume et al. [112] reported that the OH radical yield showed a six-fold decrease for PEG-coated gold nanoparticles compared to naked nanoparticle. Zavestovskaya et al. [51] showed that PEG-coated boron NP could cause more ROS generation under proton beams. Nakayama et al. [18] showed that surface coating of titanium peroxide NPs with PAA resulted in increased ROS generation by peroxidation of the coating with H2O2, leading to further ROS production in response to X-ray irradiation in the cells. The citric and malic surface coatings of NPs were proposed to be reactive owing to the net positive charge, facilitating catalysis and ROS production [52]. Klein et al. [105] showed that irradiated citric-coated iron oxide NPs generate significantly higher ROS than uncoated iron oxide NPs. Other study [26] reported the increased ROS production induced by radiation into cells treated with silica-coated iron oxide NPs in comparison to those treated with uncoated iron oxide NPs. Silica NPs have been reported as a radiosensitizer through the enhancement of t the mitochondrial ROS production [111], which means that using of silica as a coat for iron oxide NPs may facilitate their radiotherapeutic impact. Tiopronin-coated gold NPs induced necrosis by enhancing ROS production after 24-h exposure in HeLa and L929 fibroblast cells. Cheng et al. [23] hypothesized that a slight electronegative charge on the surface of gold NPs interacts with superoxide radicals/anions and catalyzes the production of ROS in vitro. The hydrophobicity of the surface coating has also been shown to be a determining factor in the induction of ROS, since the most hydrophobic coatings tend to produce the greatest/highest levels of ROS [42]. Zhu et al. [82] found a significant reduction in the level of antioxidant enzymes including catalase (CAT), superoxide dismutase (SOD), and glutathione (GSH) in the presence of galactose-PEG-coated gold NPs, indicating an increase in free radicals in HepG2 cells irradiated by/with 8Gy of 6 MV X-ray. Gilles et al. [112] showed that the coating layer may reduce the number of free radicals produced during the radiosensitization process. They demonstrated a decrease in ROS generation with PEG-coated gold NPs compared with non-capped and citrate-coated gold NPs. They also indicated that PEG coating leads to a decrease in cellular uptake of gold NPs, suggesting that stable surface coatings of the NPs disrupt the interface between the metal NP and oxygen-based molecules in the environment by scavenging ROS via chemical interactions with alcohols and thiols on the surface of the NPs [113]. Also, Singh et al. [97] reported a significant decrease in the ROS generation in samples containing silica-coated manganese NPs compared to the uncoated NPs. It seems that the coating layer could mostly affect the chemical process of ROS production rather than physico-chemical process. In spite of numerous studies reporting that coated metallic NPs can generate more ROS, several studies suggested that the coating layer could reduce the ROS generation. More studies are required to understand intricacies of coated NP’s radiosensitivity effects with respect to physico-chemical and chemical processes and biochemistry.

## The biologic responses of coated NPs

### Cell cycle alteration

Synchronizing cancer cells during a radiosensitive cell cycle phase has been recognized as an important way to enhance radiotherapy's clinical efficacy [114]. Different cell cycle phases present differential radiation sensitivity with late S-phase cell being the most radioresistant and late G2 and mitosis being the most sensitive [115]. In this context, Kai et al. [116] showed that the treatment of BEL-7402 cells with carbon-coated Fe nanoparticles caused G2/M cell cycle arrest. Alzahrani et al. [117] reported that SnO_2_ nanoparticles could inhibit the cell cycle at G0/G1 stage. Turner et al. [118] reported that metallic materials could arrest cells at the G2/M phase. Besides, glucose-coated gold NPs have been shown to cause radiosensitization by cell cycle regulation [12, 54]. Zhang et al. [119] and Roa et al. [12] reported that glucose-coated gold NPs trigger activation of the CDK kinases, leading to the accumulation of cancer cells in the G2/M phase. Zhu et al. [59] demonstrated that galactose-PEG-coated gold NPs arrested approximately 27% of cells in the G2/M phase which is more than that of naked gold NPs. Consistently, Wang et al. [10] demonstrated that the addition of glucose-coated gold NPs arrested A549 cells at the G2/M phase. Roa et al. [12] showed that irradiation following the treatment with glucose-coated gold NPs induced an increase of prostate cancer DU-145 cells in the G2/M phase and a decrease of cells in the G0/G1 phase when compared with the irradiated cells without NPs. Královec et al. [120] demonstrated a G1 and G2/M phase accumulation of HK-2 cells in presence of silica coated iron oxide nanoparticles. Li et al. [121] showed that gold nanoparticles causing cell cycle arrest is highly dependent on the surface biocompatibility of gold nanoparticles. They indicated the coating of gold nanoparticles with BSA resulted in the inhibition of lysosome rupture ability, microtubule stabilization, and a switch to G2/M arrest. However, some studies have also pointed out that NPs have no significant effect on cell-cycle distribution [122–124]. Therefore, further studies are necessary to elucidate contradictions regarding the effects of NPs on the cell cycle.

### Cell proliferation

In-vitro investigation showed that the PEG-coated gold NPs of 4.6 and 6.1 nm could decrease the cell survival rates of both cancer cell lines EMT-6 and CT26 [20]. According to Zhu et al. [125] a linear dose-dependent clonogenic survival of HepG2 cells was reported in the presence of galactose-PEG-coated and uncoated gold NPs. They showed that the survival fraction decreased by an increase of radiation dose (0–8 Gy 6MeV) in the presence of galactose-PEG-coated gold NPs was more than that of uncoated gold NPs which is mainly due to more amount of coated gold NPs uptake compared to uncoated. Zhang et al. [21] observed a radiation dose-dependent decline in a clonogenic surviving fraction in HeLa cells loaded with PEG-coated gold NPs With the same amount of PEG coating, the toxicity of small gold NPs was higher than large gold NPs. Also based on their results clonogenic survival curve was linearly decreased in the presence of coated gold NPs and non-linear in the absence of NPs which is in good agreement with the results reported by Wang et al. [10], Khoshgard et al. [61], Nakayama et al. [18] and Tu et al. [126]. Other studies reported the non-linear dose-dependent clonogenic survival decrease for different cells with and without coated NPs irradiated with photons [1, 22, 54, 58, 63, 79, 82, 127, 128].

Preliminary in vitro assays confirmed that the silica coating layer significantly reduced cellular toxicity as proved by an increase in cell viability [97]. Singh et al. [97] demonstrated that coating of metallic NPs with silica layer increase the cell viability of MC3T3-E1 cells. Klien et al. [52] confirmed that aminosilanized silica NPs enhanced the cell viability of normal (3T3) and cancer cells (MCF-7) as compared to uncoated NPs. Fathy et al. [26] demonstrated that with different doses of radiation (0, 0.5, 1, 2 and 4 Gy) of 6MV electron beams, the viability of silica-coated iron oxide NPs is less than that of uncoated iron oxide NPs for MCF-7 cancer cells. Aytac et al. [73] indicated that the cell viability ratios with polyethyleneimine-coated gold NPs are more than that of cells without NPs after 2 Gy irradiation of 6MV X-rays for L929, DLD-1 and H1299 cell lines. DEF for DLD-1 cells was 1.23 and for H1299 cells was 2.21 with 0.25 µg/ml concentrations of polyethyleneimine-coated gold NPs. Further study by Li et al. [57] reported higher cell viability of PEG-coated ceria NPs than that of irradiated alone and naked ceria NPs groups at the at doses of 0–20 Gy of ^60^Co-gamma rays for L-02 cells. Their results indicated the radioprotective effect of metallic NP coating for human normal liver cells (L-02). Cell viability of irradiated (6 and 10 MV photon beams and 6 and 12 MeV electron beams) breast cancer cells (MCF-7) in the presence of PEG-coated bismuth oxide NPs was reported to be less than cell viability in the lack of the NPs [82]. A combination of citrate-coated nickel nanoparticles and ionizing radiation (1 and 3 Gy of 60Co-gamma rays) results in a five-fold decline in cell viability, which increases radiotherapy efficacy against breast cancer cells [16]. No toxicity was reported for normal fibroblast cells [16]. Comparison of radiation sensitivity in cancers and nonmalignant cells showed that a combination of X-ray irradiation and glucose-coated gold NPs leads about 40% decrease of cell viability in cancer cells but no significant changes were observed in normal cells [14]. While, in the lack of NPs the cell viability of nonmalignant cells was less than cancerous cells indicating that presence of NPs play a key role in targeted radiation therapy. In another study, PEGylated gold NPs of 4.6 nm and 6.1 nm suppressed the cell viability rates in EMT-6 and CT 26 cell lines [129]. Soleymanifard et al. [55] demonstrated that combination of irradiation and glucose coated gold NPs dramatically decreased cell viability [55]. Compared to the irradiation alone treatment, the intracellular uptake of glucose-coated gold NPs increased cell proliferation inhibition by 30.48% for 90 kVp and 26.88% for 6 MV irradiation [11]. Compared to radiation alone, the intracellular uptake of glucose-coated gold NPs contribute to increased inhibition of cell proliferation by 64.1% and 38.7% for MCF7 cells, and 64.4% and 32.4% for QU-DB cells by 100 kVp and 6 MV X-rays, respectively [55]. In addition, the 26.8% of inhibition of cell proliferation was reported by Roa et al. [12] for DU-145 cells treated with glucose-coated gold NPs irradiated with Cs-137 source. Zhu et al. [59] manifested that galactose-coated gold NPs could result in more cell proliferation than uncoated gold NPs.

### Cell death

Increasing evidences suggest that apoptosis has a determining role in radiosensitivity. Flow cytometry analysis showed a remarkable increases in apoptosis of A549 cells by a combination of glucose-coated gold NPs and 6 MV X-ray in comparison to naked gold NPs [10]. It was found that 4.8 and 46.6 nm PEG-coated gold NPs caused rapid cell death, while 12.1 and 27.3 nm PEG-coated gold NPs induced radioenhancement by both necrosis and apoptosis [21]. Nakayama et al. [18] showed increased apoptosis in the irradiated cells loaded with PPA-coated titanium NPs. Furthermore, cells treated with combination of irradiation (X-ray) and bovine serum albumin (BSA)-coated gold NPs experienced significantly higher apoptotic induction than those treated with single treatment with X-ray [126]. Compared to bare gold NPs and radiation alone, HepG2 cells treated with galactose-PEG-GNPs showed substantial DNA double-strand breaks and apoptosis after exposure to 6MV X-rays irradiation and, thereby achieving better radiosensitization effects [59]. Song et al. [56] reported that both gold and glucose-coated gold NPs can increase cell death rate in HeLa cells after X-ray irradiation even at a lower dose. However, their results showed that the radiosensitizing effect of glucose-coated gold NPs was not as effective as uncoated gold NPs, 8% vs. Li et al. [57] showed that the total number of apoptotic cells of normal liver cells that irradiated with 20 Gy gamma-rays decreased in the presence of PEG-coated ceria NPs compared to the uncoated ceria NPs. According to these findings, PEG-coated ceria NPs could protect healthy cells more effectively against irradiation-induced damage.

### DNA damage

DNA damage occurs when highly positive NPs bind to negatively charged DNA. Comet assay confirmed an increased level of DNA damage as a result of combination of the 6 MV photons with glucose-coated NPs [55]. Measurement of γ-H2AX foci levels in cells provides a sensitive and reliable method for quantitation of the radiation-induced DNA damage response [130]. Joh et al. [79] reported an increased amount of γ-H2AX foci for irradiated (4 Gy of 150 kVp) U251 cells loaded with PEG-coated gold NPs compared to the irradiated cells without NPs. Chithrani et al. [125] showed that HeLa cells treated with 50 nm citrate-coated gold NPs increased the number of γ-H2AX and 53BP1 foci at both 220 kVp and 6 MV energies that are indicative of delayed DNA repair, a key mode of radiosensitization. Zhu et al. [59, 124] reported increased formation of γ-H2AX foci in cells treated with galactose-PEG-coated gold NPs compared to cells treated with naked gold NPs. This implies increased DNA double-strand breaks. Pancreatic cancer is relatively resistant to radiotherapy[131]. Using the MIAPaCa-2 human pancreatic cancer cell line, Nakayama et al. showed that the number of γ-H2AX foci increased significantly with a combination of radiation (30 Gy of 150 keV) and PPA-coated titanium NPs than radiation alone. The elevated levels of DNA damage may be directly related/attributed to mitochondrial dysfunction manifested as increased oxidation and loss of membrane potential which ultimately inhibit cell proliferation [59].

## Conclusion

The two-edged behavior of NP coating must be considered in design of coated nanostructures to comprise between physical and chemo-biological dose enhancement. The radiosensitization potential of coated NPs is confirmed by numerous studies but in most of them the coating layer is mostly applied to reduce toxicity of the NPs and for stability and biocompatibility aims. While the direct effects of the coating layer in radiosensitization was ignored and not considered. Going forward, knowing the physical and chemical role of the coating layer in the efficacy of radiosensitization could encourage the researchers to consciously select and apply coating layer on the NPs depending on the irradiation condition and NP type to cause more dose enhancement. Finally, more studies are necessary to directly elucidate the effects of coating materials on the radiosensitization potential of NPs compared to the noncoated NPs.

## Data Availability

The datasets used and/or analyzed during the current study are available from the corresponding author on reasonable request.

## Abbreviations

NP: Nanoparticle
ROS: Reactive oxygen species
MV: Mega voltage
KV: Kilo voltage
PEG: Polyethylene glycol
DEF: Dose enhancement factor
TEM: Transmission electron microscopy
